# Antibiotic Resistance of *Acinetobacter* Isolated in a Spanish Veterinary Teaching Hospital

**DOI:** 10.3390/ani16121768

**Published:** 2026-06-08

**Authors:** Carlota Martínez-Torrecilla, Marta E. García, Marta Pérez-Sancho, Laura Torre-Fuentes, Marta Hernández, María Ugarte-Ruiz, Julio Álvarez, Jose L. Blanco

**Affiliations:** 1 Department of Animal Health, Faculty of Veterinary Medicine, Complutense University of Madrid, 28040 Madrid, Spain; camart30@ucm.es (C.M.-T.); megarcia@ucm.es (M.E.G.); maperezs@ucm.es (M.P.-S.); 2 Veterinary Teaching Hospital, Complutense University of Madrid, 28040 Madrid, Spain; 3 VISAVET Health Surveillance Centre, Complutense University of Madrid, 28040 Madrid, Spain; ltorre04@ucm.es (L.T.-F.); maria.ugarte@ucm.es (M.U.-R.); 4 Faculty of Human Medicine, University of Valladolid, 47003 Valladolid, Spain; marta.hernandez.perez@uva.es

**Keywords:** *Acinetobacter*, antibiotic, animals, veterinary, antimicrobial resistance, gene, isolate, multidrug resistance

## Abstract

*Acinetobacter* is one of the most relevant pathogenic and nosocomial bacteria in human medicine. However, in veterinary medicine, particularly in Spain, there are very few studies on the impact and frequency of infections due to this genus. The main objective of this study was to characterise *Acinetobacter* isolates recovered at the Complutense Veterinary Teaching Hospital of the Complutense University of Madrid (HCV-UCM), with special emphasis on detecting antimicrobial resistance. A total of 23 isolates obtained from different animal species and samples over a 25-year period were included in the study, based on their identification as *Acinetobacter* by VITEK-2. Identification was made by using MALDI-TOF, VITEK-2, whole-genome sequencing (WGS) and a chromogenic medium. WGS confirmed that 13 of the 23 isolates belonged to *Acinetobacter* spp. Antimicrobial susceptibility was interpreted according to CLSI guidelines using the Kirby–Bauer disk diffusion and broth microdilution method. The proportion of clinical isolates identified as *Acinetobacter* spp. at the HCV-UCM was 0.3%. Of these, 15.4% (2/13 isolates) were classified as multidrug-resistant. Two isolates with the highest MIC for tigecycline carried the *tet(X)* gene, and two isolates harboured mutations in both *gyrA* and *parC* QRDR regions. The results of this study suggest that, in this hospital, antimicrobial resistance among *Acinetobacter* isolates may not yet be widespread.

## Simple Summary

*Acinetobacter* is a group of bacteria associated with infections in hospitalised patients. However, its current importance in veterinary practices is unclear. Pet owners are taking the health of their animals more seriously and demand higher standards of care and safety in veterinary clinics and hospitals. Consequently, tracking the presence of certain bacteria in these centres has a clear relevance for animal health. This study aimed to determine the distribution of *Acinetobacter* in a veterinary teaching hospital in Spain. We used four different identification methods, and two tests were performed to assess antimicrobial susceptibility. The results showed a low isolation frequency and a low level of antimicrobial resistance of *Acinetobacter*. Nevertheless, because animals can act as reservoirs of *Acinetobacter*, continuous monitoring of these types of bacteria is recommended.

## 1. Introduction

*Acinetobacter* spp. are Gram-negative bacilli or coccobacilli bacteria belonging to the Moraxellaceae family [1]. Among these, *A. baumannii* is one of the most clinically significant species in human medicine [2].

These bacteria are ubiquitous; they can be found in soil, water and sediment and act as commensals in both healthy humans and animals [3].

*A. baumannii* are part of the skin and upper respiratory tract microbiota in healthy individuals. The skin and mucous membranes of up to 40% of healthy adults are estimated to be colonised with this species [4].

Expression of certain factors, such as pili, outer-membrane porins, phospholipases, proteases, lipopolysaccharides, capsular polysaccharides, protein secretion systems, iron-chelating systems, penicillin-binding proteins and PER-1 β-lactamases, can confer an antimicrobial resistance phenotype to *Acinetobacter* spp. [5]. Furthermore, these species can form biofilms on biotic and abiotic surfaces, allowing them to persist in hospital environments, remaining metabolically inactive in the deepest layers to survive [4,6]. A positive correlation between biofilm formation and antibiotic resistance has been reported [4].

Other interesting *Acinetobacter* species of increasing clinical relevance are *A. pittii* and *A. nosocomialis* [6,7].

In human medicine, nosocomial infections caused by *A. baumannii* have increased over the past twenty years. This pathogen is a primary cause of sepsis, pneumonia, meningitis and endocarditis in intensive care and burn units, second only to methicillin-resistant *S. aureus* (MRSA) [8]. In hospital environments, transmission occurs through direct contact with people and contaminated fomites (e.g., reusable medical equipment, pillows, sheets, gloves, etc.) [2]. Risk factors for infection include the use of venous and urinary catheters, prolonged hospitalisation and recent surgeries [4]. Mortality rates among vulnerable patients can reach 60% due to lack of effective therapeutic options for pneumonia and bacteraemia [4,6,7,8]. The treatment of choice in case of pneumonia is carbapenems combined with other antibiotics, such as nebulised colistin [8].

In veterinary medicine, studies about frequency of infections and clinical significance of *Acinetobacter* spp. are scarce, although *A. baumannii* is considered an emerging pathogen [4] and has been isolated from a wide range of animal species [7]. Similar to what has been observed in human medicine, a high fatality rate (50–70%) has been described in veterinary patients [7,9].

The situation in companion animals (mainly hospitalised dogs and cats) is the most concerning, since isolation of the same *Acinetobacter* strain in pets and their owners suggests that pets may act as reservoirs for humans because of their close contact [3,4,10]. For instance, an investigation of two *A. baumannii* outbreaks in a veterinary hospital in the Netherlands found both clusters were related to RUH-134 (the human European clone II reference strain), and a SNP-based analysis suggested that the ancestral lineages of the two outbreaks appeared in the 1980s [11].

The European Committee on Antimicrobial Susceptibility Testing (EUCAST) indicates that members of the genus *Acinetobacter* are naturally resistant to the following antibiotics: ampicillin, amoxicillin, amoxicillin-clavulanic acid, cefotaxime, ceftriaxone, aztreonam, ertapenem, trimethoprim, tetracycline, doxycycline and fosfomycin [12]. The antibiotic groups active against this genus are carbapenems, polymyxins, fluoroquinolones and aminoglycosides [6,7,8].

While carbapenems are the antibiotics of choice for treating *A. baumannii* infections, the emergence of carbapenem-resistant strains has already been described [4]. Consequently, carbapenem-resistant *A. baumannii* has been classified as a “Critical Group” pathogen in the latest World Health Organization (WHO) Bacterial Priority Pathogen List [13]. In addition, strains resistant to all antibiotic groups have also been described, including β-lactams, aminoglycosides, cephalosporins, carbapenems, fluoroquinolones, trimethoprim/sulfamethoxazole and recently colistin (although the mechanisms behind this resistance are not fully studied yet) [8].

For instance, a study from a veterinary clinical hospital in South Africa found that 95% of isolates were resistant to at least one antibiotic, and 60% were classified as multidrug-resistant (MDR) [14].

According to the “One Health” concept, human, animal and ecosystem health is closely interrelated. Therefore, any changes in the relationships between these three elements may increase the risk of appearance of new diseases. It is, therefore, of paramount importance to characterise the dynamics of bacterial species shared between animals and humans. Approximately 60% of emerging infectious diseases in humans originate from animals [15]. In addition, there is a lack of studies on *Acinetobacter* spp. in veterinary medicine, particularly in Spain.

Because of this, we performed an investigation to assess the epidemiological situation and the relative frequency of isolation of *Acinetobacter* spp. infection in animals visiting the Veterinary Clinical Hospital of the Complutense University of Madrid (HCV-UCM). Moreover, we studied antimicrobial resistance, focusing on finding possible MDR bacterial strains.

## 2. Materials and Methods

First, all *Acinetobacter* suspected strains found among all bacterial isolates stored at the Microbiology and Parasitology Laboratory at the HCV-UCM from 1998 to 2024 were identified. Of the total collection of 4560 isolates, 27 isolates had been classified as *Acinetobacter* spp. using the VITEK-2 system.

These 27 frozen isolates were subcultured onto blood–agar plates (BIOMÉRIEUX, Paris, France) and incubated at 37 °C for 24–48 h. Four isolates did not grow after multiple attempts; therefore, twenty-three presumptive *Acinetobacter* isolates were successfully recovered. We used these 23 isolates for the following analyses, with the earliest isolate dating from 2009 and the most recent isolate dating from 2024.

Table 1 provides detailed information on these 23 isolates.

Second, successfully recovered isolates were subjected to bacterial identification using MALDI-TOF and whole-genome sequencing (WGS). Identification was performed in accordance with the manufacturer’s protocols; however, only MALDI-TOF scores above 2.000 were considered valid for the assignment of *Acinetobacter* species. In addition, isolates were cultured on CHROMagar^TM^ *Acinetobacter* (Chromagar, Paris, France), a specific and differential chromogenic medium for *Acinetobacter* species.

Third, antimicrobial susceptibility testing was performed using two methods, the Kirby–Bauer disk diffusion method and the broth microdilution method. The Kirby–Bauer method was performed and interpreted according to the Clinical & Laboratory Standards Institute (CLSI), specifically, the M100 document [16].

Briefly, the Kirby–Bauer disk diffusion method was performed on Mueller–Hinton agar, with six antibiotics tested per plate. A total of 24 antibiotics from different pharmacological groups were evaluated: amoxicillin-clavulanic acid (OXOID; 30 µg), amikacin (OXOID; 30 µg), ampicillin-sulbactam (OXOID; 20 µg), cefotaxime (BIO-RAD; 30 µg), cefepime (BIO-RAD; 30 µg), cefotixin (BIO-RAD; 30 µg), ceftazidime (BIO-RAD; 30 µg), chloramphenicol (OXOID; 30 µg), erythromycin (OXOID; 10 µg), gentamicin (OXOID; 10 µg), imipenem (OXOID; 10 µg), levofloxacin (OXOID; 5 µg), meropenem (OXOID; 10 µg), polymyxin B (OXOID; 300 iu), rifampicin (OXOID; 5 µg), temocillin (OXOID; 30 µg), tetracycline (OXOID; 30 µg) and tigecycline (OXOID; 15 µg). These antibiotics were selected based on the literature, antibiotics most frequently used according to the HCV-UCM protocol and those included in prior related studies.

The broth microdilution method was performed according to ISO 20776-1:2019 [17] guidelines. Minimum inhibitory concentrations (MICs) for amikacin (AMI), sulfamethoxazole (SMX), trimethoprim (TMP,) ciprofloxacin (CIP), tetracycline (TET), meropenem (MERO), azithromycin (AZI), nalidixic acid (NAL), chloramphenicol (CHL), cefotaxime (FOT), tigecycline (TGC), ceftazidime (TAZ), colistin (COL), ampicillin (AMP) and gentamicin (GEN) were determined in a selection of isolates using the two-fold broth microdilution reference method following commercial panel EUVSEC3 (Trek Diagnostic Systems; Thermo Scientific, Waltham, MA, USA). Interpretation of the quantitative data was carried out according to the guidelines of CLSI [16].

Last, bacterial genomic DNA was extracted and purified using Qiagen DNA Blood and Tissue Kit (Hilden, Germany), following the manufacturer’s instructions, and quantification of the DNA concentration was performed using a Qubit^®^ fluorometer (Invitrogen-Therm Fisher Scientific; Waltham, MA, USA). WGS libraries were prepared with the Nextera XT DNA Library Preparation Kit (Illumina, San Diego, CA, USA) following manufacturer instructions. The concentrations of each library were adjusted to 4 nM to obtain equimolar DNA concentrations in a single pool of libraries and sequenced on a MiSeq platform (Illumina, San Diego, CA, USA).

Raw reads obtained from sequencing were filtered out with Trimmomatic [18] and evaluated with FastQC [19]. Reads that passed the quality control were characterised using Kraken [20].

Reads from isolates identified as *Acinetobacter* were assembled with SPAdes [21], and the quality of the assemblies was evaluated with QUAST [22] and CheckM [23]. Assemblies were screened for the presence of antibiotic resistance genes with AMRFinder v3.11.2 with database version 2022-12-19.1 [24].

## 3. Results

### 3.1. Acinetobacter Isolation

VITEK-2 initially identified 23 isolates (0.5% = 23/4560) as *Acinetobacter* spp. These presumptive *Acinetobacter* isolates were isolated from respiratory samples (6/23 = 26.1%), wound and incision samples (4/23 = 17.4%), ocular samples (3/23 = 13%), skin samples (2/23 = 8.6%), otic exudates (2/23 = 8.6%), soft tissue (2/23 = 8.6%), uterine fluid (1/23 = 4.3%), urine (1/23 = 4.3%), cerebrospinal fluid (1/23 = 4.3%) and a lymph sample (1/23 = 4.3%) (Table 1).

Among the 23 isolates included in this study, 5/23 were from cats (21.7%), 5/23 were from dogs (21.7%), 4/23 were from horses (17.4%), 3/23 were from tortoises (13%), 1/23 was from a sheep (4.2%), 1/23 was from a mouse (4.2%), 1/23 was from a rabbit (4.2%), 1/23 was from a cow (4.2%), 1/23 was from a chameleon (4.2%), and 1/23 was from a bird (4.2%) (Table 1).

### 3.2. Acinetobacter Identification

Table 2 shows the comparison of the results obtained using the four identification methods applied in the study: MALDI-TOF, VITEK-2, WGS and chromogenic medium. MALDI-TOF, VITEK-2 and WGS can identify isolates at the species level, whereas the chromogenic medium provides a qualitative assessment of the possible growth of *Acinetobacter* spp. Whole-genome sequences generated in this project are available at NCBI “https://www.ncbi.nlm.nih.gov/sra/PRJNA1455583” (accessed on 21 April 2026), and additional information on these isolates is provided in the Supplementary Material (Table S2).

According to the WGS results, 13 isolates (56.5% = 13/23) were confirmed as *Acinetobacter* spp., 5 out of which were *A. baumannii* (Table 2). MALDI-TOF identified as belonging to the *Acinetobacter* genus 12 (52.2%) of the 23 isolates, almost half of which would be *A. baumannii* (5/23). VITEK-2 identified the bacterial growth as *Acinetobacter* spp. in 100% of the isolates and 21.7% (5/23) as *A. baumannii* species. Finally, the chromogenic medium found positive growth compatible with *Acinetobacter* spp. in 78.2% of the isolates (18/23).

MALDI-TOF was the identification method with the highest concordance with WGS, reaching 92.3% of observed agreement at the genus level (12/13) and 46.2% at the species level among the *Acinetobacter* spp. isolates (6/13). Agreement was higher for VITEK-2 when considering identification of isolates as belonging to the *Acinetobacter* genus (100%), but much lower when considering identification of *A. baumannii* (30.8%). In the case of the chromogenic medium, a 92.3% concordance with WGS was found when considering the genus level. Results from all four identification methods agreed in 38.5% of the isolates identified as *Acinetobacter* spp. by WGS (5/13).

Therefore, the percentage of sample types and animal species previously described for the 23 presumptive *Acinetobacter* isolates should now be interpreted as follows for the 13 confirmed *Acinetobacter* isolates. These 13 verified *Acinetobacter* were isolated from ocular samples (3/13 = 23.1%), exudate samples (3/13 = 23.1%), soft tissue (2/13 = 15.4%), uterine fluid (1/13 = 7.7%), shell wounds (1/13 = 7.7%), cerebrospinal fluid (1/13 = 7.7%), lymph (1/13 = 7.7%), wounds and abscesses (1/13 = 7.7%) (Table 1).

Among these 13 isolates, 3/13 were from dogs (23.1%), 2/13 were from horses (15.4%), 2/13 were from tortoises (15.4%), 1/13 was from a sheep (7.7%), 1/13 was from a cat (7.7%), 1/13 was from a mouse (7.7%), 1/13 was from a cow (7.7%), 1/13 was from a bird (7.7%), and 1/13 was from a chameleon (7.7%) (Table 1).

### 3.3. Acinetobacter Antibiotic Resistance

**Figure 1:**
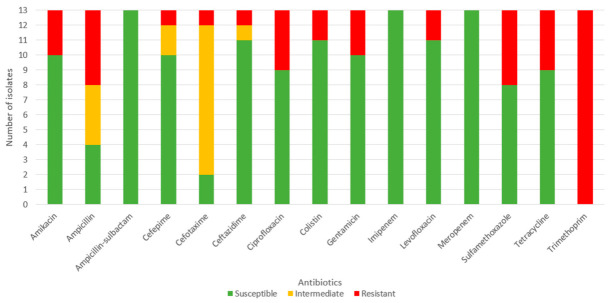
Antimicrobial resistance study from 13 confirmed *Acinetobacter* isolates. Isolates were classified as resistant (red colour), intermediate (yellow) or susceptible (green) depending on the diameter of the inhibition zone (Kirby–Bauer method) or the established breakpoint (broth microdilution method) for each antibiotic.

The number of isolates with a resistant phenotype for each antibiotic is shown in Figure 1. The number of resistant isolates ranged between 4 and 6, with higher levels of resistance (13/13) for trimethoprim and lower for carbapenems (full susceptibility).

As shown in Figure 1, the phenotype for certain antibiotics (amoxicillin-clavulanic acid, azithromycin, cefotixin, chloramphenicol, erythromycin, nalidix acid, polymyxin B, rifampicin, temocillin, tigecycline) cannot be interpreted due to the lack of specific breakpoints in the CLSI M100 document [16] for the *Acinetobacter* genus.

The following mechanisms linked to AMR were identified in the 13 *Acinetobacter* isolates: amvA, msr(E), mph(E), mef(F), msr(G), nreB, tet(X3), adeC, tet(B), tet(39), sul2, sul1, merE, merD, merA, merT, merR, aph(6)-Id, aph(3″)-Ib, aac(6′)-Ib4, aph(3′)-Vla, ant(3″)-IIa, aac(3)-IIe, aac(3)-IVa, ant(2″)-Ia, aac(6′)-Ian, aac(3)IId, abaF, qacEdelta1, arr-3, blaADC variants (blaADC166, blaADC26, blaADC76, blaADC165), several blaOXA variants (blaOXA106, blaOXA64, blaOXA144), blaCARB-16, blaBRO, dfrA44, parC_S84L, gyrA_S81L, adeS_H189Y, l floR, ars(B) and dfrA40. The 13 *Acinetobacter* isolates carried between 1 and 24 AMR mechanisms (mean = 7.3 mechanisms/isolate).

Table 3 shows the antibiotic resistance genes organised according to the associated antibiotic.

Certain genes, such as *blaOXA* and *blaADC* variants, were found in *Acinetobacter* as expected. Other beta-lactamase-encoding genes, which could have been acquired horizontally (*blaCARB-16*, *blaBRO*), were also found; however, no carbapenemase-associated genes were detected. Of note, the two isolates with the highest MIC for tigecycline (8 µg/mL and 1 µg/mL compared to 0.5 or <0.25 for the rest) carried the *tet(X)* gene, and two isolates had mutations in both *gyrA* and *parC* QRDR regions (both with a resistance phenotype to CIP and NAL). In addition, between 17 and 24 AMR markers were detected in two isolates (Table A1).

A table providing a more detailed association between each resistance gene/mechanism gene and the phenotypic susceptibility of each strain is presented in the Supplementary Material (Table S1).

## 4. Discussion

A total of 23 isolates obtained from different animal species and samples over a 25-year period were included in the study, initially identified as *Acinetobacter* spp. by VITEK-2. But, out of the 23 isolates initially considered as putative *Acinetobacter*, 13 were confirmed as *Acinetobacter* based on the WGS results.

These 13 isolates constituted the subject of the present study. The very low proportion of clinical isolates presumptively identified as *Acinetobacter* spp. (0.5%), coupled with the even lower proportion of confirmed *Acinetobacter* spp. isolates (0.3%) indicates that the clinical relevance of this genus in animals visiting the HCVC is limited, in agreement with a previous study also describing a low frequency (6.5%) of isolation of *Acinetobacter* spp. in dogs and cats in Reunion Island [10] or in an Italian veterinary teaching hospital [25]. However, other studies based on syndromic surveillance described higher prevalence levels of *Acinetobacter* spp. infection in dogs (16.3%) and cats (12%) [9].

These discrepancies suggest that the true incidence of this genus in hospitals and veterinary clinics remains poorly defined and may even vary depending on the country and type of surveillance applied.

The majority of *Acinetobacter* isolates were recovered from ocular samples (3/13 = 23.1%), exudate samples (3/13 = 23.1%) and soft tissue (2/13 = 15.4%). These results differ from those reported by Van der Kolk [7]. They observed that, in dogs, *Acinetobacter* was most commonly isolated from pyoderma, chronic eczema, systemic and local infections, vaginal or saliva samples and urinary infections, whereas in cats it was most frequently isolated from intravenous catheters, necrotizing fasciitis, septic shock, or liver biopsies. This diversity of results highlights the great opportunistic capacity of *Acinetobacter*, which can be isolated from any type of sample.

Regarding the animal species from which *Acinetobacter* was isolated, our results are consistent with the literature, with the majority of the isolates originating from companion animals, mainly dogs and cats (4/13 = 30.8%), compared to the rest of isoltes [10].

Regarding the bacterial identification and considering the WGS as the gold standard, MALDI-TOF was the most reliable method, with a high level of agreement with WGS considering the identification of isolates as *Acinetobacter* spp. (92.3%). At the species level, MALDI-TOF correctly identified 46.2% (6/13) of *Acinetobacter* isolates, yielding a more limited discriminatory power. Performance was lower for VITEK-2. The limited ability of MALDI-TOF to identify *Acinetobacter* at the species-level identification has also been reported in other studies [26,27,28], which concluded that this method is highly reliable for identifying *A. baumannii*, but WGS is recommended for the remaining species. The chromogenic medium could be recommended as a rapid and effective first-line screening tool since it showed a near-complete agreement with WGS (92.3%).

Fifteen antibiotics were selected for the broth microdilution method (some of which were also included in the disk diffusion test) as this panel is routinely used in our laboratory for *Enterobacteriaceae* surveillance. It included most of the antimicrobials of interest for comparison with the genomic data.

*Acinetobacter* has intrinsic resistance to multiple antibiotics, including several that are part of the EUVSEC3 plate and disk diffusion test (ampicillin, amoxicillin-clavulanic acid, cefotaxime, trimethoprim and tetracycline); consequently, they were not considered to classify the strains as MDR.

Despite these intrinsic resistances, several susceptible and intermediate strains to these antibiotics have been described.

For example, 30.7% (4/13 isolates) were susceptible, and 38.5% (5/13 isolates) were intermediate to ampicillin; 15.4% (2/13 isolates) were susceptible, and 84.6% (11/13 isolates) were intermediate to cefotaxime; and 69.2% (9/13 isolates) were susceptible to tetracycline. Similar findings have also been reported in other studies, such as those by Benga [3], Hamouda [29], or Sebola [14], in which susceptibility to tetracycline was observed.

Based on these findings, two questions can be formulated: Why do certain bacterial strains harbour antibiotic resistance genes but are susceptible to those antibiotics? Why do other bacterial strains without detectable resistance genes show phenotypic resistance?

Eight confirmed *Acinetobacter* isolates (61.5%) exhibited resistance to at least one antibiotic. This result is lower than that reported in the study by Sebola et al. [14], in which 95% of the isolates in their veterinary teaching hospital were resistant to at least one antibiotic.

Overall, high levels of antibiotic resistance were not observed in our study, with more than half of the isolates remaining susceptible to all antibiotics tested, except for ampicillin, cefotaxime and trimethoprim.

Resistance to aminoglycosides (amikacin and gentamicin), fluoroquinolones (ciprofloxacin, levofloxacin), sulphonamides (sulfamethoxazole), tetracyclines (tetracycline) and polymyxins (colistin) was found in 2–5 isolates. We want to emphasise the resistance to colistin, since it is considered a last-resort antibiotic for human use and is classified in the “B category” according to the European Medicines Agency (EMA) [30]. Resistance to colistin is especially worrying, as it is a last-resort therapeutic option in human medicine also for treating specifically MDR *Acinetobacter* infections [4].

We considered MDR bacteria as those non-susceptible to at least one antibiotic in three or more pharmacological classes [31].

In the present study, 15.4% of isolates were classified as MDR (2/13 isolates). This proportion is lower than previously reported in the literature (60%) [14], but it is still above the ideal scenario in which the prevalence of MDR bacteria is close to zero.

While a large variety of AMR mechanisms was detected in 1 to 7 of the 13 sequenced *Acinetobacter* isolates, some of them were expected, considering the previous description in this genus of carriage of certain resistance mechanisms such as beta-lactamase-encoding genes [32]. Still, finding the *tet(X)* gene, even at a low frequency (2/13 isolates: strain “1125” from 2009 and strain “4255” from 2022), is a more concerning result, particularly given the increased MIC observed especially in one of the two *tet(X3)-*carrying isolates. Previous studies have described the dissemination of *tet(X3)*-carrying plasmids in *Acinetobacter* of animal origin [33], further highlighting the risk of horizontal spread of such MGE in “One Health” settings. Similarly, the presence of QRDR mutations in CIP and NAL-R *A. baumannii* isolates, also previously found at a higher frequency in clinical collections [34], should also be further considered.

Our study shows the low rate of *Acinetobacter* spp. isolation in animal processes. Of the 23 isolates first considered to be of this species, only 13 were ultimately confirmed as *Acinetobacter* spp. by WGS. Clearly, this low rate of isolation is one of the limitations of this work, along with other factors, such as the lack of clinical information or the fact that some of these isolates were incidental findings and not the true source of infection, which is supported by the low resistance rate among these isolates and the fact that no animal died as a result of the infection.

## 5. Conclusions

Nowadays, health and welfare of companion animals are increasingly prioritised. As a result, owners are demanding the highest standard of medical care for their pets, seeking veterinary hospitals that guarantee superior quality and safety services. For this reason, studies conducted in national reference hospitals, where the clinical impact and epidemiological significance of specific opportunistic bacteria remain unknown, are of paramount importance.

Our findings suggest that, in the hospital under study, antimicrobial resistance is not yet widespread among *Acinetobacter* isolates of veterinary origin. Nevertheless, as animals can act as reservoirs for resistant strains, continuous and systematic monitoring of antimicrobial susceptibility by validated methods is recommended.

## Figures and Tables

**Table 1:** Strain identification, host species, date of primary isolation and sample type of the 23 isolates.

Strain Identification	Host Species	Date of Primary Isolation	Sample
1125	Horse	14 September 2009	Corneal cotton swab
1156	Horse	22 October 2009	Skin cotton swab
1409	Dog	31 May 2010	Otic exudate
1308	Tortoise	17 January 2011	Tracheal exudate
1643	Horse	29 November 2014	Abdomen incision
1963	Horse	05 May 2016	Uterine fluid
2080	Sheep	25 October 2016	Soft tissue
2724	Dog	26 September 2018	Skin and hair
3321	Galapagos tortoise	17 November 2020	Exudate
3386	Cat	02 March 2021	Soft tissue
3534	Cat	28 June 2021	Urine
3556	Mouse	12 July 2021	Ocular cotton swab
3627	Cat	11 October 2021	Tracheal lavage
3684	Tortoise	03 November 2021	Shell wound cotton swab
3815	Cat	11 February 2022	Tracheal lavage
3904	Dog	24 March 2022	Wound exudate
4062	Rabbit	14 September 2022	Nasal sample
4070	Cat	22 September 2022	Tracheal lavage
4255	Cow	22 December 2022	Cerebrospinal fluid
4338	Cockatoo	13 March 2023	Lymph
4348	Dog	13 March 2023	Wound and abscess
4485	Chameleon	12 June 2023	Conjunctive exudate
4676	Dog	25 September 2024	Otic exudate

**Table 2:** Identification of 23 isolates obtained using MALDI-TOF, VITEK-2, WGS and chromogenic medium.

ID of the Isolates	WGS	MALDI-TOF	VITEK-2	^1^ CHROMOGENIC MEDIUM
1125	*A. pitti*	*Acinetobacter* spp.	*Acinetobacter* spp.	+
1156	No identification	^2^ No identification	*A. iwoffii*	+
1308	No identification	*Alcaligenes faecalis*	*A. iwoffii*	+
1409	*A. baumannii*	*A. baumannii*	*Acinetobacter* spp.	+
1643	No identification	No identification	*Acinetobacter* spp.	+
1963	*A. baumannii*	*A. baumannii*	*A. baumannii*	+
2080	*A. indicus*	No identification	*A. iwoffii*	+
2724	*Moraxella catarrhalis*	*Moraxella canis*	*Acinetobacter* spp.	−
3321	*A. bereziniae*	*Acinetobacter* spp.	*A. iwoffii*	+
3386	*A. radioresistens*	*A. radioresistens*	*A. radioresistens*	+
3534	*Solibacillus silvestris*	*Solibacillus silvestris*	*A. iwoffii*	+
3556	*A. baumannii*	*A. baumannii*	*A. baumannii*	+
3627	*Neisseria* spp.	*Neisseria flavescens subflava*	*A. iwoffii*	−
3684	*Acinetobacter* spp.	*Acinetobacter* spp.	*A. iwoffii*	−
3815	*Neisseria* spp.	No identification	*A. iwoffii*	−
3904	*A. calcoaceticus*	*Acinetobacter* spp.	*A. baumannii*	+
4062	*Staphylococcus hominis*	*Staphylococcus hominis*	*Acinetobacter* spp.	+
4070	*Glasserella parasuis*	*Staphylococcus hominis*	*A. iwoffii*	−
4255	*A. iwoffii*	*A. pseudolwoffii*	*A. iwoffii*	+
4338	*A. baumannii*	*A. baumannii*	*A. baumannii*	+
4348	*A. baumannii*	*A. baumannii*	*A. baumannii*	+
4485	*A. bereziniae*	*Acinetobacter* spp.	*A. iwoffii*	+
4676	*Moraxella catarrhalis*	*Moraxella canis*	*A. iwoffii*	+

^1^ The “+” sign indicates the growth of pink bacterial colonies, which means a possible growth of *Acinetobacter* species, and the “−“ sign indicates the growth of blue bacterial colonies, suggestive of the growth of something different to *Acinetobacter*. ^2^ “No identification” is usually associated with the absence of isolate information in the MALDI-TOF database or with a very different bacterial spectrum.

**Table 3:** Antimicrobial resistance mechanism described in 13 *Acinetobacter* isolates. The number of isolates carrying each gene is in brackets.

Antibiotic	Antibiotic Resistance Gen
Amikacin	*aph(3′)-*VIa (1), *aac(6′)-Ib4* (1), *aac(3)-Iva* (1) *aac(6′)-Ian* (1), *adeS_H189Y* (2), *aph(6)-Id* (5), *ant(3”)-Iia* (5), *aph(3”)-Ib* (4), *aac(3)-Iie* (1), *ant(2”)-Ia* (1)
Amoxicillin-clavulanic acid	*blaADC* (2), *blaADC26* (2), *blaADC76* (1), *blaADC165* (1), *blaADC166* (1), *blaOXA* (7), *blaOXA64* (2), *blaOXA106* (1), *blaOXA144* (1), *blaBRO* (0), *blaCARB-16* (0), *adeS_H189Y* (0)
Ampicillin	*blaADC* (2), *blaADC26* (2), *blaADC76* (1), *blaADC165* (1), *blaADC166* (1), *blaBRO* (0), *blaCARB-16* (0), *blaOXA* (7), *blaOXA64* (2), *blaOXA106* (1), *blaOXA144* (1), *adeS_H189Y* (0)
Ampicillin-sulbactam	*blaADC* (2), *blaADC26* (2), *blaADC76* (1), *blaADC165* (1), *blaADC166* (1), *blaOXA* (7), *blaOXA64* (2), *blaOXA106* (1), *blaOXA144* (1), *blaBRO* (0), *blaCARB-16* (0), *adeS_H189Y* (0)
Azithromycin	*msr(E)* (2), *msr(G)* (0), *mef(F)* (0), *mph(E)* (2), *amvA* (7), *adeS_H189Y* (2)
Cefepime	*blaADC* (2), *blaADC26* (2), *blaADC76* (1), *blaADC165* (1), *blaADC166* (1), *blaOXA* (7), *blaOXA64* (2), *blaOXA106* (1), *blaOXA144* (1), *blaBRO* (0), *blaCARB-16* (0), *adeS_H189Y* (0)
Cefotaxime	*blaADC* (2), *blaADC26* (2), *blaADC76* (1), *blaADC165* (1), *blaADC166* (1), *blaOXA* (7), *blaOXA64* (2), *blaOXA106* (1), *blaOXA144* (1), *blaBRO* (0), *blaCARB-16* (0), *adeS_H189Y* (0)
Cefotixin	*blaADC* (2), *blaADC26* (2), *blaADC76* (1), *blaADC165* (1), *blaADC166* (1), *blaOXA* (7), *blaOXA64* (2), *blaOXA106* (1), *blaOXA144* (1), *blaBRO* (0), *blaCARB-16* (0), *adeS_H189Y* (0)
Ceftazidime	*blaADC* (2), *blaADC26* (2), *blaADC76* (1), *blaADC165* (1), *blaADC166* (1), *blaOXA* (7), *blaOXA64* (2), *blaOXA106* (1), *blaOXA144* (1), *blaBRO* (0), *blaCARB-16* (0), *adeS_H189Y* (0)
Ciprofloxacin	*gyrA_S81L* (2), *parC_S84L* (2), *adeS_H189Y* (2)
Chloramphenicol	*floR* (2), *adeS_H189Y* (2)
Colistin	—
Erythromycin	*msr(E)* (2), *msr(G)* (0), *mef(F)* (0), *mph(E)* (2), *amvA* (7), *adeS_H189Y* (2)
Gentamicin	*aac(3)Iid* (1), *aph(3′)-*VIa (1), *aac(6′)-Ib4* (1), *aac(3)-Iva* (1), *aac(6′)-Ian* (1), *adeS_H189Y* (2), *aph(6)-Id* (5), *ant(3”)-Iia* (5), *aph(3”)-Ib* (4), *aac(3)-Iie* (1), *ant(2”)-Ia* (1)
Imipenem	*blaADC* (2), *blaADC26* (2), *blaADC76* (1), *blaADC165* (1), *blaADC166* (1), *blaOXA* (7), *blaOXA64* (2), *blaOXA106* (1), *blaOXA144* (1), *blaBRO* (0), *blaCARB-16* (0), *adeS_H189Y* (0)
Levofloxacin	*gyrA_S81L* (2), *parC_S84L* (2), *adeS_H189Y* (2)
Meropenem	*blaADC* (2), *blaADC26* (2), *blaADC76* (1), *blaADC165* (1), *blaADC166* (1), *blaOXA* (7), *blaOXA64* (2), *blaOXA106* (1), *blaOXA144* (1), *blaBRO* (0), *blaCARB-16* (0), *adeS_H189Y* (0)
Nalidixic Acid	*gyrA_S81L* (2), *parC_S84L* (2), *adeS_H189Y* (2)
Polymyxin B	—
Rifampicin	*arr-3* (1)
Sulfamethoxazole	*sul1* (1), *sul2* (5)
Temocillin	*blaADC* (2), *blaADC26* (2), *blaADC76* (1), *blaADC165* (1), *blaADC166* (1), *blaOXA* (7), *blaOXA64* (2), *blaOXA106* (1), *blaOXA144* (1), *blaBRO* (0), *blaCARB-16* (0), *adeS_H189Y* (0)
Tetracycline	*tet(B)* (2), *tet(39)* (1), *adeS_H189Y* (2), *adeC* (2), *tet(X3)* (2)
Tigecycline	*tet(B)* (2), *tet(39)* (1), *adeS_H189Y* (2), *adeC* (2), *tet(X3)* (2)
Trimethoprim	*dfrA40* (0), *dfrA44* (1)

## Data Availability

All whole-genome sequences generated in this project are available at NCBI “https://www.ncbi.nlm.nih.gov/sra/PRJNA1455583” (accessed on 21 April 2026), under the ID: PRJNA1455583. Individual information is in the Supplementary Material Table S2.

## Supplementary Materials

The following supporting information can be downloaded at https://www.mdpi.com/article/10.3390/ani16121768/s1, Table S1: Correlation between phenotypic susceptibility and resistance genes in 13 isolates; Table S2: Individualised information about the 13 *Acinetobacter* whole-genome sequences.

## Appendix A

**Table A1:** Antimicrobial resistance genes and mechanism of the 13 *Acinetobacter* isolates.

ID of Isolate	Genus and Species	Antimicrobial Resistance Genes
1125	*A. pittii*	*amvA*; *nreB*; *tet(X3)*; *sul2*, *sul1*, *merE*, *merD*, *merA*, *merT*, *merR*; *aph(6)-Id*; *aph(3”)-Ib*; *aac(3)-Iid*; *aac(6′)-Lb4*; *aph(3′)-Vla*; *abaF*; *qacEdelta1*; *arr-3*; *blaADC*; *blaOXA*; *blaCARB-16*; *msr (E)*; *mph(E)*; *drfA44*
1409	*A. baumannii*	*amvA*; *nreB*; *abaF*; *adeC*; *blaADC-166*; *blaOXA-106*; *ant(3”)-Iia*
1963	*A. baumannii*	*amvA*; *nreB*; *sul2*; *merT*; *merR*; *aph(6)-Id*; *aph(3”)-Ib*; *abaF*; *ant(3”)-Iia*; *aac(3)-Iie*; *aac(6′)-Ian*; *blaOXA-64*; *blaADC-26*; *parC_S84L*; *gyrA_S81L*; *tet(B)*; *adeS_H189Y*
2080	*A. indicus*	*sul2*; *aph(6)-Id*; *aph(3”)-Ib*; *floR*
3321	*A. bereziniae*	*sul2*; *aph(6)-Id*; *aph(3”)-Ib*; *blaOXA*; *msr(E)*; *mph(E)*; *tet(B)*; *floR*; *tet(39)*; *ant(2”)-Ia*
3386	*A. radioresistens*	*blaOXA*
3556	*A. baumannii*	*amvA*; *nreB*; *abaF*; *ant(3”)-Iia*; *blaOXA-64*; *blaADC-26*; *adeS_H189Y*
3684	*Acinetobacter* sp. WCHAc010034	*blaOXA*
3904	*A. calcoaceticus*	*amvA*; *nreB*; *blaADC*; *blaOXA*
4255	*A. iwoffii*	*tet(X3)*; *sul2*; *aph(6)-Id*; *aph(3”)-Ib*; *dfrA40*
4338	*A. baumannii*	*amvA*; *nreB*; *abaF*; *adeC*; *ant(3”)-Iia*; *parC_S84L*; *gyrA_S81L*; *blaADC-76*; *blaOXA-144*
4348	*A. baumannii*	*amvA*; *nreB*; *abaF*; *blaOXA*; *ant(3”)-Iia*; *blaADC-165*
4485	*A. bereziniae*	*blaOXA*
