# Supplementary material for: Antibiotic Resistance of Acinetobacter Isolated in a Spanish Veterinary Teaching Hospital
Source: Animals (Basel). 2026 Jun 8;16(12):1768. doi: 10.3390/ani16121768 (PMC13295950; doi:10.3390/ani16121768)
Supplement: Supplementary file 1 [file animals-16-01768-s001.zip › Table S2.pdf]

SUPPLEMENTARY MATERIAL

Table S2: Individualised information about the 13 Acinetobacter whole genome sequences.

Object ID	Corresponding URLs
49429302	https://www.ncbi.nlm.nih.gov/sra/RUN:49429302
49429301	https://www.ncbi.nlm.nih.gov/sra/RUN:49429301
49429300	https://www.ncbi.nlm.nih.gov/sra/RUN:49429300
49429299	https://www.ncbi.nlm.nih.gov/sra/RUN:49429299
49429298	https://www.ncbi.nlm.nih.gov/sra/RUN:49429298
49429297	https://www.ncbi.nlm.nih.gov/sra/RUN:49429297
49429296	https://www.ncbi.nlm.nih.gov/sra/RUN:49429296
49429295	https://www.ncbi.nlm.nih.gov/sra/RUN:49429295
49429294	https://www.ncbi.nlm.nih.gov/sra/RUN:49429294
49429293	https://www.ncbi.nlm.nih.gov/sra/RUN:49429293
49429292	https://www.ncbi.nlm.nih.gov/sra/RUN:49429292
49429291	https://www.ncbi.nlm.nih.gov/sra/RUN:49429291
49429290	https://www.ncbi.nlm.nih.gov/sra/RUN:49429290
